# Smad3 deficiency improves islet-based therapy for diabetes and diabetic kidney injury by promoting β cell proliferation *via* the E2F3-dependent mechanism

**DOI:** 10.7150/thno.67034

**Published:** 2022-01-01

**Authors:** Hong-Lian Wang, Biao Wei, Hui-Jun He, Xiao-Ru Huang, Jing-Yi Sheng, Xiao-Cui Chen, Li Wang, Rui-Zhi Tan, Jian-Chun Li, Jian Liu, Si-Jin Yang, Ronald CW Ma, Hui-Yao Lan

**Affiliations:** 1 Research Center for Integrated Medicine and Department of Nephrology, Affiliated Traditional Chinese Medicine Hospital of Southwest Medical University, Luzhou, Sichuan, 646000, China.; 2 Department of Medicine and Therapeutics, and Li Ka Shing Institute of Health Sciences, the Chinese University of Hong Kong, Hong Kong, 999077, China.; 3 Guangdong-Hong Kong Joint Laboratory on Immunological and Genetic Kidney Diseases, Guangdong Provincial People's Hospital, Guangdong Academy of Medical Sciences, Guangzhou, Guangdong, 510080, China.; 4 State Key Laboratory of Bioelectronics, Jiangsu Key Laboratory for Biomaterials and Devices, School of Biological Sciences & Medical Engineering, Southeast University, Nanjing, China.; 5 Key Laboratory of Prevention and Management of Chronic Kidney Disease of Zhanjiang City, Institute of Nephrology, Affiliated Hospital of Guangdong Medical University, Zhanjiang, Guangdong, 524001, China.; 6 National Traditional Chinese Medicine Clinical Research Base, Affiliated Traditional Chinese Medicine Hospital of Southwest Medical University, Luzhou, Sichuan, 646000, China.; 7 CUHK-Guangdong Provincial People's Hospital Joint Research Laboratory on Immunological and Genetic Kidney Diseases, the Chinese University of Hong Kong, Hong Kong, 999077, China.

**Keywords:** Smad3, islet transplantation, diabetes, E2F3, cell cycle

## Abstract

**Rationale:** Poor β cell proliferation is one of the detrimental factors hindering islet cell replacement therapy for patients with diabetes. Smad3 is an important transcriptional factor of TGF-β signaling and has been shown to promote diabetes by inhibiting β cell proliferation. Therefore, we hypothesize that Smad3-deficient islets may be a novel cell replacement therapy for diabetes.

**Methods:** We examined this hypothesis in streptozocin-induced type-1 diabetic mice and type-2 diabetic db/db mice by transplanting Smad3 knockout (KO) and wild type (WT) islets under the renal capsule, respectively. The effects of Smad3KO versus WT islet replacement therapy on diabetes and diabetic kidney injury were examined. In addition, RNA-seq was applied to identify the downstream target gene underlying Smad3-regulated β cell proliferation in Smad3KO-db/db versus Smad3WT-db/db mouse islets.

**Results:** Compared to Smad3WT islet therapy, treatment with Smad3KO islets produced a much better therapeutic effect on both type-1 and type-2 diabetes by significantly lowering serum levels of blood glucose and HbA1c and protected against diabetic kidney injuries by preventing an increase in serum creatinine and the development of proteinuria, mesangial matrix expansion, and fibrosis. These were associated with a significant increase in grafted β cell proliferation and blood insulin levels, resulting in improved glucose intolerance. Mechanistically, RNA-seq revealed that compared with Smad3WT-db/db mouse islets, deletion of Smad3 from db/db mouse islets markedly upregulated *E2F3*, a pivotal regulator of cell cycle G1/S entry. Further studies found that Smad3 could bind to the promoter of *E2F3*, and thus inhibit β cell proliferation *via* an E2F3-dependent mechanism as silencing *E2F3* abrogated the proliferative effect on Smad3KO β cells.

**Conclusion:** Smad3-deficient islet replacement therapy can significantly improve both type-1 and type-2 diabetes and protect against diabetic kidney injury, which is mediated by a novel mechanism of E2F3-dependent β cell proliferation.

## Introduction

β cell loss or deficiency is a common feature in both type-1 and 2 diabetes mellitus (T1DM and T2DM). β cell or islet transplantation is an attractive therapy and has been successfully applied in patients with T1DM. The islets or β cells used for transplantation are usually from the cadaveric donor or induced pluripotent stem cells (iPSC) 1, 2. More than 1500 cases of clinical islet transplantation have been completed in patients with T1DM following the well-established Edmonton Protocol 1. Although about 50% of patients hold insulin independence for 5 years after transplantation, engraftment failure is a common concern clinically. One of the factors that hinder islet transplantation is the extremely low proliferative rate of the grafted islet β cells 3. In addition, the hazardous stimuli from the graft microenvironment such as inflammation, immune allograft rejection, and metabolic stress may cause transplanted β cells to undergo apoptosis and necrosis, resulting in progressive graft loss 1. Therefore, improvement of the transplanted β cell proliferation is pivotal for the success of islet transplantation-based treatment on diabetes.

Transforming growth factor-beta (TGF-β) signaling plays pleiotropic roles in many biological processes. The signal transduction initiates by the binding of TGF-β ligand to its type 2 receptor TGFBR2. The latter recruits and activates type 1 receptor TGFBR1 by phosphorylation. Activated TGFBR1 further phosphorylates the receptor-regulated Smad proteins (R-Smads), Smad2 and Smad3. The R-Smads form a complex with Smad4, which translocates into the nucleus to transcriptionally regulate target genes 4, 5. It is reported that the pancreatic islet maintains universally activated TGF-β signaling as revealed by a relatively high level of phosphorylated Smad2/3 in the physiological conditions. However, in response to insulin demand, the TGF-β signaling activity is decreased, which is accompanied by increased β cell proliferation 6. In addition, pharmacological inhibition of TGF-β signaling promotes β cell proliferation *in vitro* and *in vivo* 7, 8. More importantly, deletion of Smad3 results in more robust β cell proliferation compared with the deletion of Smad2 in mice with partial pancreatectomy, indicating that Smad3 but not Smad2 is the primary effector downstream of TGF-β signaling to regulate β cell proliferation 6. Later studies show that Smad3 suppresses β cell proliferation by inducing the expression of cyclin-dependent kinase inhibitors (CDKIs), including p16, p21, and p57 7, 8.

Our recent work also found that deletion of Smad3 in db/db mice (Smad3KO-db/db) prevents the onset of overt diabetes without obesity, hyperglycemia, insulin resistance, and glucose intolerance. Interestingly, Smad3KO-db/db mice show islet hyperplasia with significantly increased β cell proliferation and persistent hyperinsulinemia 9. This implies that Smad3 is pathogenic in diabetes and targeting Smad3 in β cells may represent a novel β cell or islet replacement therapy for diabetes. This was examined in the present study by using Smad3 knockout (Smad3KO) islet transplantation in streptozocin (STZ)-induced type-1 diabetic mice and in type-2 diabetic db/db mice.

## Results

### Smad3KO islet transplantation produced a better therapeutic effect on STZ-induced type-1 diabetes

**Figure 1:**
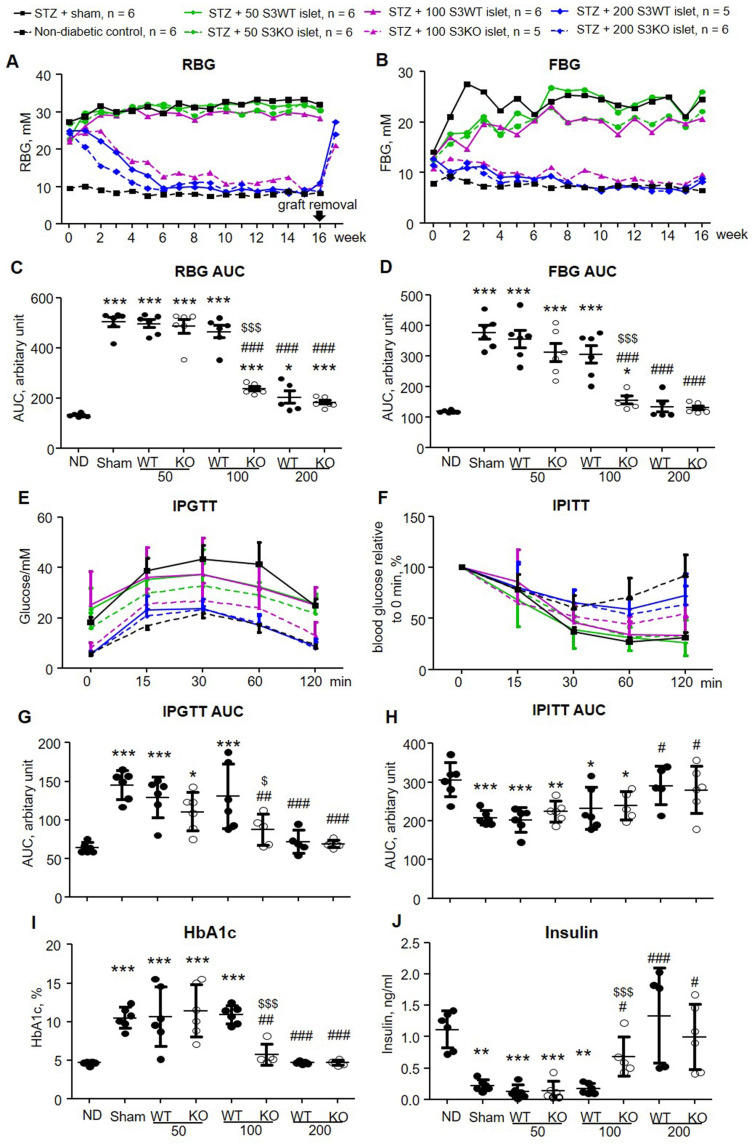
** Transplantation with Smad3KO islets shows better glycemic control and improves glucose intolerance in STZ-treated mice. (A, B)** Weekly RBG and FBG. Only the mean values of blood glucose are plotted to make the graph concise. Note that islet grafts removal at 16 weeks post-transplantation causes recurred hyperglycemia (arrow in A). **(C, D)** Quantitation of AUC (area under curve) of RBG and FBG. **(E)** Intraperitoneal glucose tolerance test (IPGTT). **(F)** Intraperitoneal insulin tolerance test (IPITT). **(G)** Quantitation of AUC for the IPGTT test. **(H)** Quantitation of AUC for the IPITT test. **(I)** Quantitation of blood level of HbA1c. **(J)** Serum insulin level determined by ELISA. Animal numbers used for islet treatment (A, B, E, and F) are indicated in the up panel of the figure. Each dot represents one animal and data are expressed as mean ± SD for C, D and G to J. *p < 0.05, **p < 0.01, ***p < 0.001 versus normal mice (ND). ^#^*p* < 0.05, ^##^*p* < 0.01, ^###^*p* < 0.001 versus diabetic mice with sham-operation; ^$^*p* < 0.05, ^$$$^*p* < 0.001 versus 100 Smad3WT islets treatment.

To compare the therapeutic efficacy of Smad3KO versus Smad3WT islet on diabetes, we firstly performed syngeneic islet transplantation under the renal capsule of STZ-induced diabetic mice where hyperglycemia is caused by β cell deficiency. Diabetic mice on day 7 after STZ injection were transplanted with 3 doses of islets (50, 100, and 200 islets per mouse) that were freshly isolated from Smad3WT or Smad3KO mice. Both random blood glucose (RBG) and fasting blood glucose (FBG) levels were monitored weekly for 16 weeks. The transplantation doses were selected based on the previous findings that transplantation of 300 islets is sufficient to restore euglycemia in β cell-depleted mice rapidly 10. As shown in Figure 1A to D, the diabetic mice with sham operation developed persistent hyperglycemia over 16 weeks. Transplantation with a low dose (50 islets) of Smad3KO or WT islets failed to reduce both RBG and FBG levels. However, diabetic mice receiving 100 Smad3KO islets, but not the same dose of Smad3WT islets, gradually recovered the normal RBG and FBG similar to those receiving 200 Smad3KO or WT islets. We also found that the blood-glucose-lowering effect is solely attributed to the transplanted islets as hyperglycemia recurred immediately once the islet-bearing kidney was removed from 16 weeks onwards (arrow, Figure 1A).

Impaired glucose tolerance was also developed in the sham-operated diabetic mice, which was significantly relieved by transplanting the high dose (200) of Smad3KO or WT islets and 100 Smad3KO islets, but not by the low dose of 50 Smad3KO or WT islets and 100 Smad3WT islets (Figure 1E and G). As expected, STZ-induced diabetic mice didn't develop insulin resistance.

**Figure 2:**
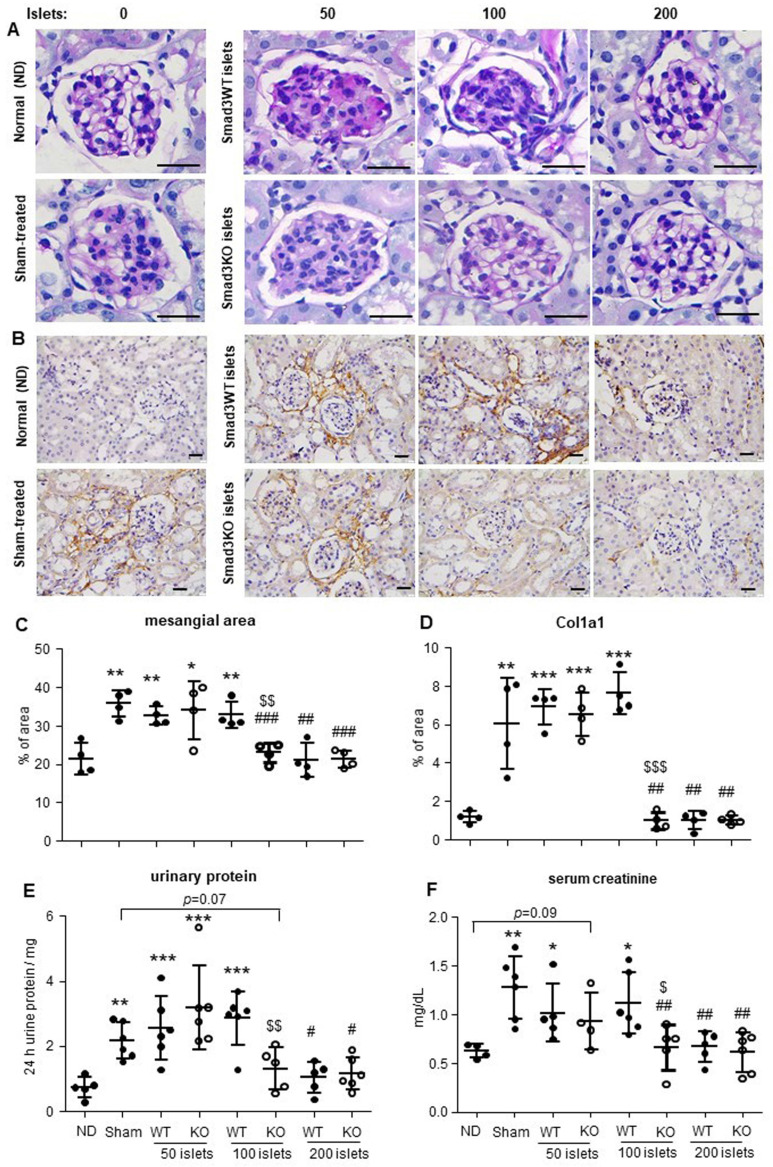
** Effect of Smad3KO or WT islet transplantation on renal injury in STZ-induced diabetic mice at week 16 post-transplantation. (A)** Periodic Acid-Schiff (PAS) staining shows the glomerular mesangial expansion. **(B)** Immunohistochemistry detects the expression of Col1a1. **(C)** Quantitation of the glomerular mesangial area as shown in panel A. **(D)** Quantitation of the Col1a1 expression as shown in panel B. **(E)** Determination of 24 h total urinary protein. **(F)** Quantitation of serum creatinine. Each dot represents one animal. ND, non-diabetic normal control. *p < 0.05, **p < 0.01, and ***p < 0.001 versus ND group. #*p* < 0.05, ##*p* < 0.01, and ###*p* < 0.001 versus sham group. ^$^*p* < 0.05, ^$$^*p* < 0.01, and ^$$$^*p* < 0.001 versus Smad3WT 100 group. Scale bar, 25 μm.

In contrast, prolonged response to insulin was observed in hyperglycemic mice with sham-operation or ineffective therapy with 50 Smad3WT or KO islets and 100 Smad3WT islets (Figure 1F and H). In line with the recovered euglycemia and improved glucose intolerance, diabetic mice giving 200 Smad3KO or WT islets and 100 Smad3KO islets, but not 50 Smad3KO or WT islets and 100 Smad3WT islets, showed decreased blood HbA1c level accompanied by increased insulin level (Figure 1 I and J). Furthermore, STZ-induced diabetes caused growth retardation, which was partially recovered in mice transplanted with 200 Smad3KO or WT islets and 100 Smad3KO islets (Figure S1). In addition, STZ-induced diabetic mice also developed diabetic nephropathy at 16 weeks as demonstrated by mesangial expansion, interstitial fibrosis, proteinuria, and increased serum creatinine (Figure 2). Intriguingly, treatment with 100 Smad3KO islets, but not 100 Smad3WT islets, resulted in protection against the development of diabetic kidney disease, although this was also notable in diabetic mice treated with the high dose of 200 Smad3KO or WT islets (Figure 2). These findings demonstrate a better therapeutic efficacy of Smad3KO islets on STZ-induced diabetes and suggest about 50% reduction in the islets needed to achieve competent glycemic control and protection against diabetic kidney injury by using Smad3-deficient islet replacement therapy for diabetes.

### Smad3KO islet transplantation produced a better therapeutic effect on type-2 diabetes in db/db mice

**Figure 3:**
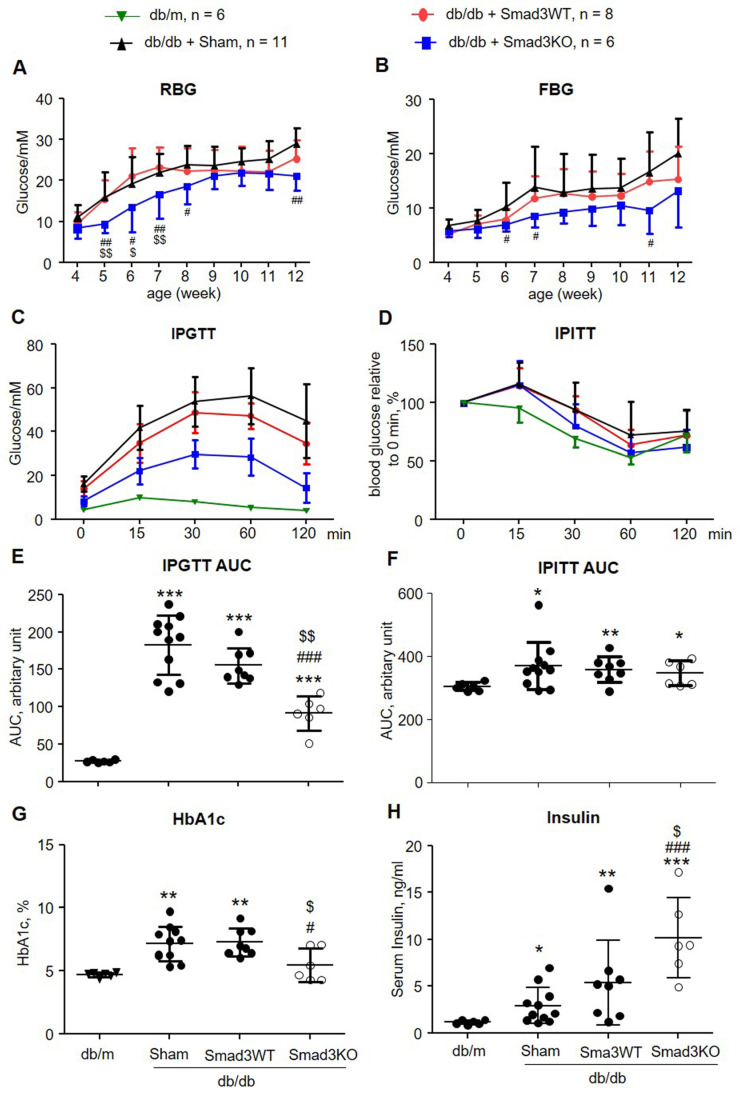
** Transplantation with Smad3KO islets attenuates hyperglycemia and glucose intolerance in db/db mice. (A, B)** Weekly RBG and FBG; **(C, E)** IPGTT and its AUC quantitation. **(D, F)** IPITT and its AUC quantitation. **(G)** Levels of HbA1c. **(H)** Serum levels of insulin. Animal numbers used for islet treatment (A to D) are indicated in the up panel of the figure. Each dot represents one animal for E to H. Data are expressed as mean ± SD. *p < 0.05, **p < 0.01, ***p < 0.001 versus age-matched db/m mice. ^#^*p* < 0.05, ^##^*p* < 0.01,^ ###^*p* < 0.001 versus sham-db/db mice. ^$^*p* < 0.05, ^$$^*p* < 0.01 versus db/db mice receiving Smad3WT islets.

**Figure 4:**
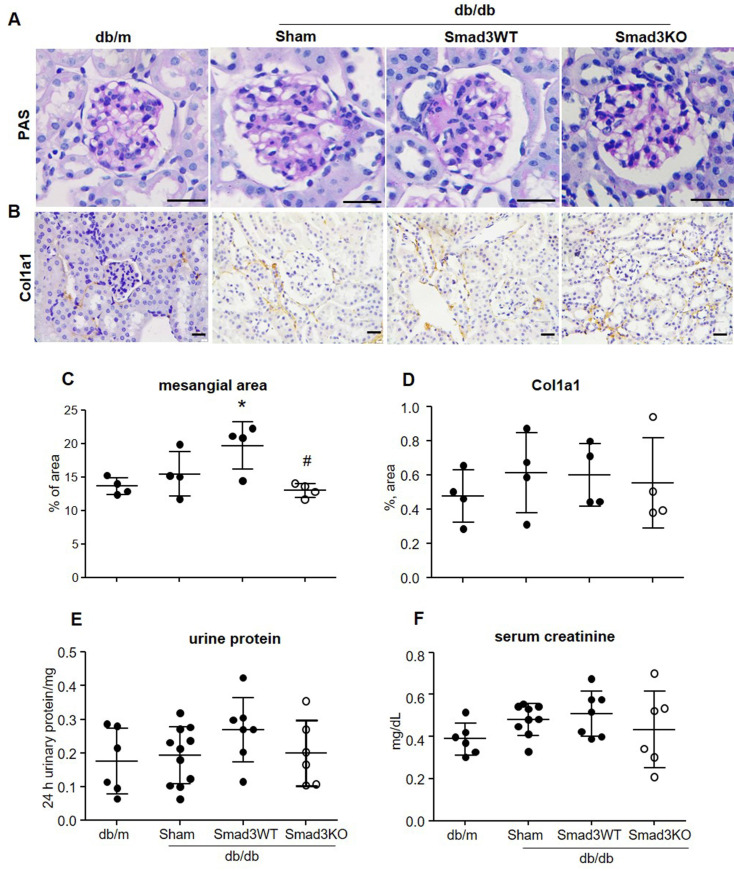
** Effect of Smad3KO and WT islet transplantation on renal injury in db/db mice at the age of week 12 (8 weeks treatment over week 4 to 12). (A)** Periodic Acid-Schiff (PAS) staining shows the glomerular mesangial expansion. **(B)** Immunohistochemistry detects expression of Col1a1. **(C)** Quantitation of the glomerular mesangial area as shown in panel A. **(D)** Quantitation of the Col1a1 expression as shown in panel B. **(E)** Determination of 24 h total urine protein. **(F)** Quantitation of serum creatinine. Each dot represents one animal. * p < 0.05 versus age-matched db/m mice. #*p* < 0.05 versus Smad3WT db/db mice. Scale bar, 25 μm.

It has been reported that islet transplantation improves T2DM in a high fat diet-fed mouse model challenged with a low dose of STZ 11. We next investigated whether transplantation of Smad3KO islets also produces a better therapeutic effect on T2DM by treating db/db mice with 250 Smad3KO or WT islets from the pre-diabetic age of week 4 to week 12. We found that transplantation with either Smad3KO or WT islets did not influence the growth of recipient db/db mice (Figure S2). Although db/db mice receiving Smad3WT islet transplantation showed a trend decrease of RBG and FBG compared with the sham-operated db/db mice, db/db mice transplanted with the equivalent dose of Smad3KO islets showed a significant decrease in RBG and FBG (Figure 3A and B). Glucose intolerance developed in db/db mice was significantly improved by Smad3KO but not Smad3WT islet transplantation, although insulin resistance was not altered (Figure 3C-F). Consistently, compared with Smad3WT islet transplantation or sham-operation, db/db mice treated with Smad3KO islet transplantation also presented a significant decrease in blood HbA1c levels, which was accompanied by a notable increase in levels of blood insulin (Figure 3G and H). Furthermore, treatment with 250 Smad3KO islets significantly attenuated glomerular mesangial expansion (Figure 4). However, since no significant changes in serum creatinine, proteinuria, and renal fibrosis were evidenced in db/db mice at the age of 12 weeks, no obvious alterations in these parameters were found in db/db mice treated with Smad3KO or WT islets (Figure 4). Collectively, these data again indicate that transplantation of Smad3KO islets produces a better treatment over Smad3WT islets in type-2 diabetic db/db mice.

### Smad3 deficiency promotes β cell proliferation in the grafted islets in both STZ-induced diabetic mice and db/db mice

**Figure 5:**
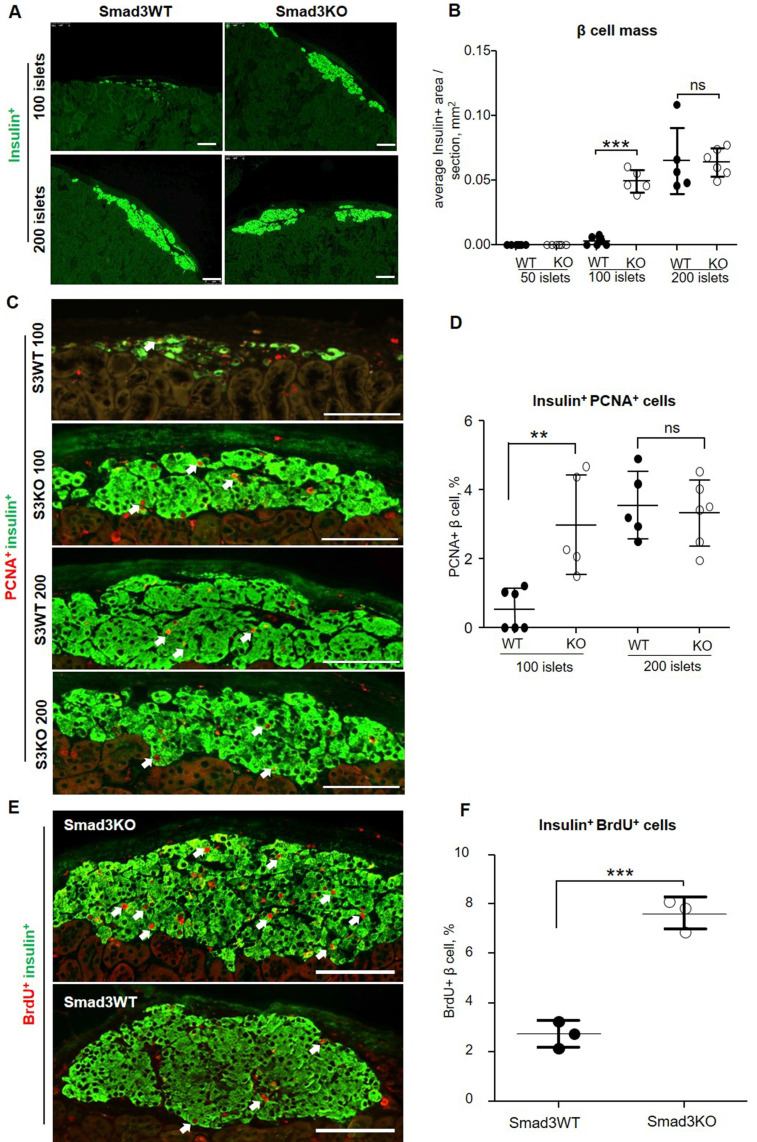
** Smad3 deficiency promotes β cell proliferation in islets grafted into STZ-induced diabetic mice. (A)** 16 weeks post-transplantation, grafted islet β cells are revealed by immunostaining for insulin. **(B)** Quantitation of the index of average β cell area per section as described in the Methods.** (C, D)** Double immunostaining and quantitation of PCNA^+^ insulin^+^ β cell (arrows) in grafted islets under renal capsule at week 16 after islet transplantation. **(E, F)** Early β cell proliferation on day 7 post-transplantation of 100 Smad3KO or Smad3WT islets revealed by BrdU^+^ insulin^+^ cells (arrows). Each dot represents one animal and data are expressed as mean ± SD. ** p< 0.01 and ***p < 0.001. Scale bar, 100 μm.

We next investigated the potential mechanisms whereby transplantation of Smad3KO islets produces a better therapeutic effect on both T1DM and T2DM. We firstly examined the β cell mass in the grafted islets by immunostaining with insulin. In STZ-induced diabetes, we failed to detect the existence of insulin-positive cells under the renal capsule in mice engrafted with 50 Smad3KO or WT islets at week 16 after transplantation, indicating the loss of β cells. Unexpectedly, diabetic mice receiving 100 Smad3WT islets showed only a few dispersed insulin-producing β cell clusters, but this was largely increased in diabetic mice treated with 100 Smad3KO islets, resulting in nearly a 17-fold increase in the β cell mass (Figure 5A and B). Interestingly, diabetic mice giving a high dose of 200 Smad3KO or WT islets exhibited a similar β cell mass (Figure 5A and B). These findings were consistent with the dose-dependent effect of islet transplantation on hyperglycemia as shown in Figure 1A and B.

We next investigated whether the increased β cell mass in Smad3KO islet grafts is associated with enhanced β cell proliferation. Two-color immunofluorescence showed that at week 16 post-islet transplantation, the mice treated with 100 Smad3WT islets showed undetectable or rare PCNA^+^ insulin^+^ cells, which was largely increased in those receiving 100 Smad3KO islets or a high dose of 200 Smad3KO or WT islets (Figure 5C and D). We then investigated whether Smad3 controls the early β cell proliferation in the grafted islets under progressive diabetic conditions by BrdU labeling on day 7 post-islet transplantation when levels of blood glucose remained high. BrdU labeling revealed a 3-fold increase in BrdU^+^ insulin^+^ β cells in 100 Smad3KO islet grafts compared with the same dose of Smad3WT islet grafts (Figure 5E and F). This finding indicates that Smad3 deficiency largely promotes β cell proliferation through the S-phase cell cycle under hyperglycemic conditions, contributing to a 17-fold increase in the β cell mass as seen at week 16 post-islet transplantation (Figure 5A and B). Taken together, all these findings suggest that Smad3 deficiency largely promotes β cell proliferation, thereby restoring euglycemia in STZ-induced type-1 diabetes.

**Figure 6:**
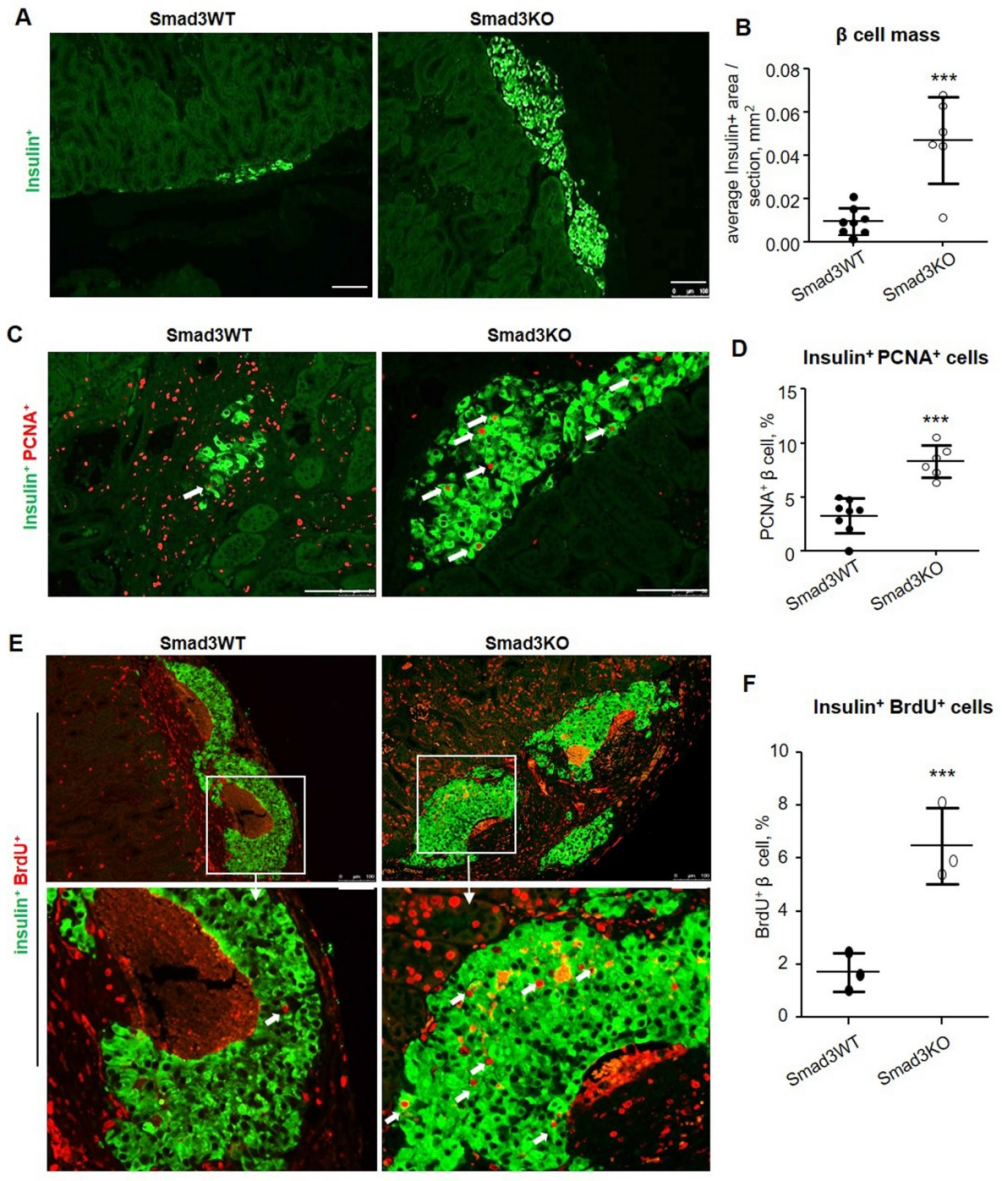
** Smad3 deficiency promotes β cell proliferation of islet grafts in db/db mice. (A, B)** 8 weeks post-transplantation, β cells in the islets-bearing kidney were detected by the anti-insulin antibody and quantitated in serial sections as described in the Methods. **(C, D)** Two-color immunostaining and quantitation of PCNA^+^ insulin^+^ β cells (arrows) in grafts of Smad3KO and WT islets at 8 weeks after islet transplantation. **(E, F)** Two-color immunostaining and quantitation of BrdU^+^ insulin^+^ proliferative β cells (arrows) in grafts of Smad3KO and WT islets on day 7 post-transplantation in 12-week-old db/db mice. The images in the lower panel are the enlarged view of corresponding images in the upper panel as indicated. Scale bar, 100 μm. ***p < 0.001 compared with Smad3WT islet grafts.

Similarly, high β cell proliferative activities were also detected in Smad3KO islet grafts in type-2 diabetic db/db mice as shown by a 5-fold increase in the β cell mass when compared to those with Smad3WT islet grafts at the age of week 12 after islet transplantation (Figure 6A and B), which was associated with a 2.5-fold increase in insulin^+^ PCNA^+^ cells (Figure 6C and D). By using BrdU-labelling, we also detected a 3-fold increase in insulin^+^ BrdU^+^ β cells in Smad3KO islet grafts on day 7 post-transplantation in 12-week-old db/db mice (Figure 6E and F). This finding again confirms that β cells lacking Smad3 are resistant to hyperglycemia and remain highly proliferative to maintain the β cell mass to improve T2DM.

### Smad3 deficiency promotes β cell proliferation *in vitro*

**Figure 7:**
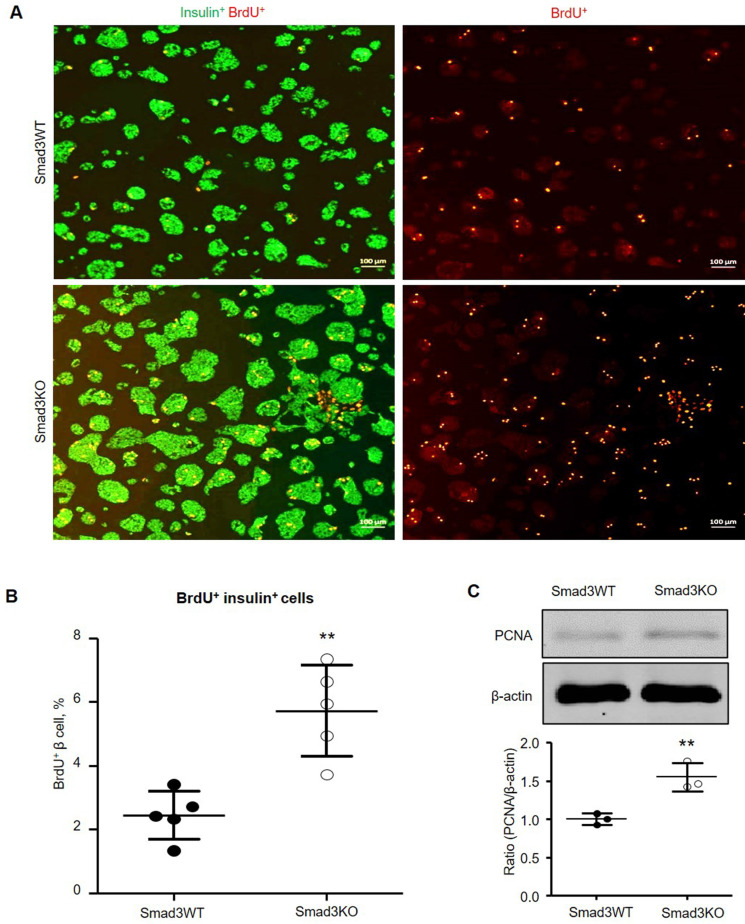
** Smad3 deficiency promotes β cell proliferation in cultured islet cells. (A)** Smad3KO or WT islet cells were cultured for 72 h with BrdU labeling in the last 24 h. Double immunostaining detects BrdU^+^ insulin^+^ β cells. Scale bar, 100 μm. **(B)** Quantitation of BrdU^+^ insulin^+^ β cells. **(C)** Western blot analysis of PCNA expression in cultured islet cells. Each dot represents one randomly selected field and data are expressed as mean ± SD for 5 independent studies. **p < 0.01 compared with Smad3WT islet culture. Scale bar, 100 μm.

It has been reported that pharmacological inhibition of TGFBR1 promotes β cell proliferation *in vitro* 7, 8. We, therefore, investigated whether the genetic deletion of *Smad3* also promotes β cell proliferation by culturing the Smad3KO and WT islet cells followed by BrdU labeling. Double immunofluorescence showed that islet cells lacking Smad3 exhibited a significantly higher level of BrdU-positive β cells (5.7%) compared with Smad3WT islet cells (2.4%) (Figure 7A and B). The augmented proliferation of Smad3KO β cells was also demonstrated by increased expression of PCNA as revealed by Western blot analysis (Figure 7C), confirming the key regulatory role of Smad3 in β cell proliferation.

### RNA-seq identifies *E2F3* as a Smad3 target gene that regulates β cell proliferation

**Figure 8:**
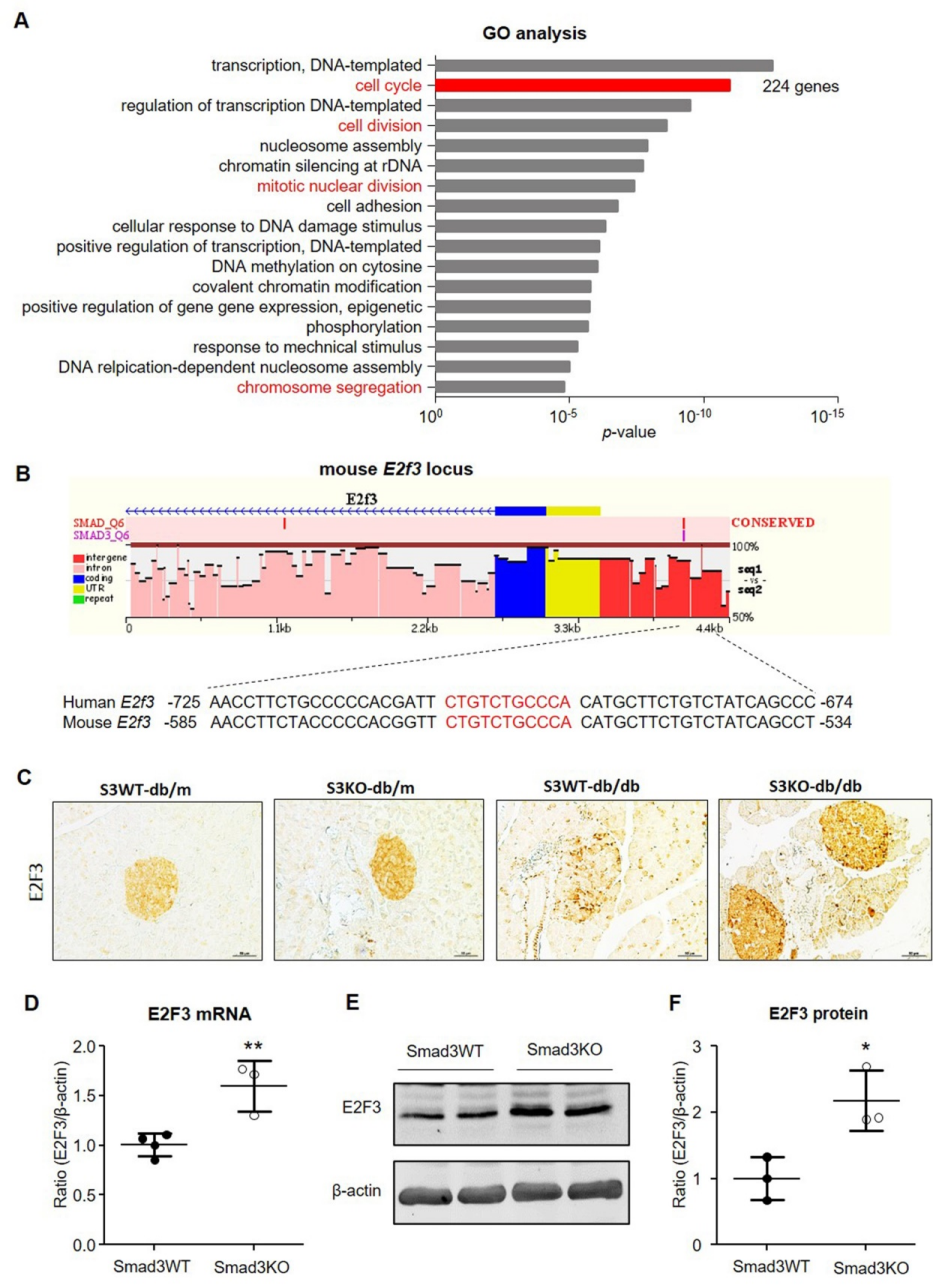
** Smad3 deficiency upregulates E2F3 expression in islets *in vivo* and *in vitro*. (A)** GO analysis of DEGs in islets between Smad3WT-db/db and Smad3KO-db/db mice by RNA-seq. Genes with differential expression of equal or more than 2 folds are considered as DEGs (both upregulation and downregulation). Cell proliferation-related GO terms are marked in red. The total number of genes in the term of cell cycle is indicated on the right. **(B)** Cartoon to show the Smad3 binding site (nucleotides in red) on mouse *E2F3* locus predicted by ECR browser software.** (C)** Immunohistochemistry detects the expression of E2F3 in pancreatic islets from mice of indicated genotypes. Scale bar, 50 μm. **(D-F)** Smad3KO and Smad3WT islet cells were cultured for 48 h followed by the analysis of *E2F3* mRNA by RT-PCR and protein by Western blot. Each dot represents one independent cell culture and data are expressed as mean ± SD. *p < 0.05, **p < 0.01 compared with Smad3WT islet culture.

To uncover the downstream mechanism of Smad3 in regulating β cell proliferation, we performed RNA-seq in islets isolated from db/m or db/db mice with or without deletion of *Smad3* gene including Smad3WT-db/db, Smad3KO-db/db, Smad3WT-db/m, and Smad3KO-db/m mice as previously described 9. Pathway enrichment analysis was conducted among the differentially expressed genes (DEGs) between Smad3WT and Smad3KO islets in db/m or db/db background. In the genetic background of db/db, Smad3KO resulted in substantial changes of cell cycle-related genes or pathways as revealed by GO and KEGG analysis (Figure 8A and Figure S3). In view of the individual genes, RNA-seq analysis revealed an increase in expression of genes involving the cyclin, cyclin-dependent kinases (CDKs), and cell division cycle (CDC) protein family in Smad3KO-db/db islets compared with Smad3WT-db/db islets, which was further validated in cultured Smad3KO islet cells by RT-PCR (Figure S4 A and C). In contrast, in the genetic background of db/m, Smad3KO caused no remarkable influence on cell cycle-related genes or pathways (Figure S3B and C). It is reported that TGF-β/Smad3 signaling negatively regulates the cell cycle by inducing the cyclin-dependent kinase inhibitor genes (CDKIs) 7, 8, 12. Surprisingly, none of these CDKI genes' expression was suppressed in Smad3KO-db/db islets *in vivo* and in cultured Smad3KO islet cells as revealed by RNA-seq and RT-PCR, respectively (Figure S4B and C). In contrast, the expression levels of several CDKI genes like p16, p18, and p57 were even upregulated in cultured Smad3KO islet cells (Figure S4C). This implies that an alternative mechanism exists in Smad3-regulated β cell proliferation.

We thus analyzed the Smad3 binding potential for the promoter sequences of the 224 DEGs in the GO term of cell cycle with the ECR browser software (Figure 8A). Among them, one gene named *E2F3* gained our attention as it's a key and terminal executor regulating G1/S entry of the cell cycle 13, 14. We found a potential Smad3 binding site in the promoter of mouse *E2F3* locus which is also conserved in human (Figure 8B).

**Figure 9:**
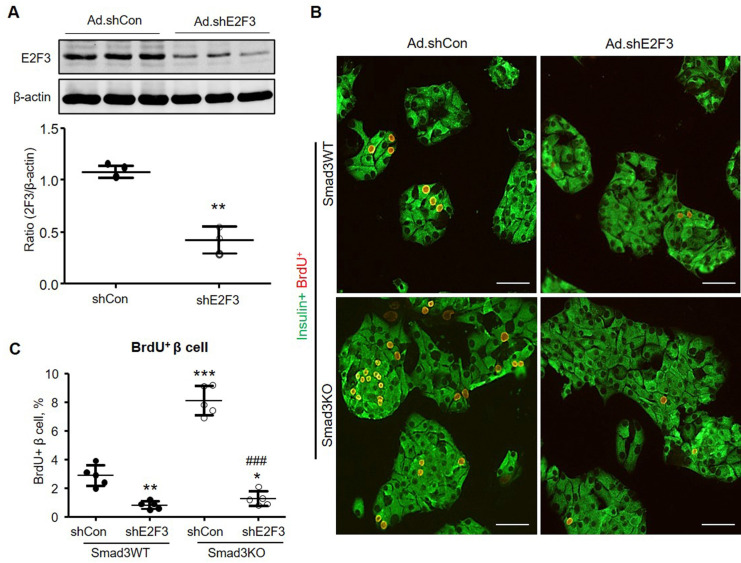
** Silencing *E2F3* abolishes the augmented β cell proliferation in cultured Smad3KO islet cells. (A)** Dissociated Smad3WT islet cells were cultured in the presence of Ad-shE2F3 or Ad-shCon virus for 48 h. E2F3 expression was detected by Western blot. n = 3 culture replicates in each group. **p < 0.01 compared with Ad.shCon. **(B, C)** Dissociated Smad3WT and Smad3KO islet cells were cultured in the presence of Ad-shE2F3 or Ad-shCon virus for 48 h followed by BrdU labeling for another 24 h. β cell proliferation was shown by double immunostaining for insulin^+^ BrdU^+^ cells (red nuclei). Each dot represents one independent experiment and data are expressed as mean ± SD. Scale bar, 100 μm. **p < 0.01, ***p < 0.001 versus Smad3WT shCon. ^###^*p* < 0.001 versus Smad3KO Ad.shCon.

To investigate whether E2F3 is essential for Smad3-regulated β cell proliferation, we examined the association between E2F3 and Smad3 in islets. Consistent with the RNA-seq, immunohistochemistry detected that the expression of E2F3 was largely reduced in the islets of Smad3WT-db/db mice but greatly increased in Smad3KO-db/db mice (Figure 8C). Furthermore, increased E2F3 expression was also observed in cultured Smad3KO islet cells at both RNA and protein levels (Figure 8 D-F). In contrast, adenovirus-mediated silence of *E2F3* abrogated the proliferative activity of β cells (insulin^+^ BrdU^+^) in Smad3KO islet cells (Figure 9), revealing an essential role for E2F3 in Smad3-regulated β cell proliferation.

**Figure 10:**
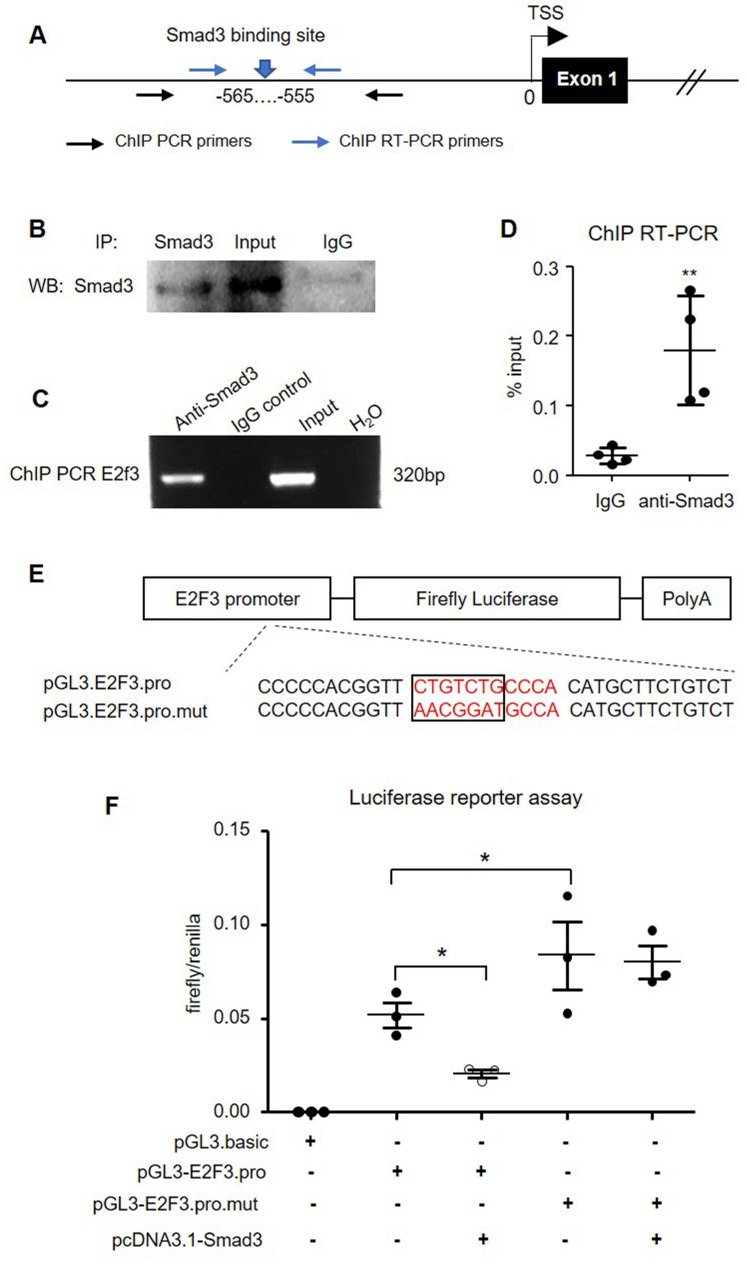
** Smad3 negatively regulates *E2F3* transcription by binding to its promoter. (A)** A map position of primers for ChIP PCR and ChIP RT-PCR. **(B)** Immunoprecipitation in mouse islets to show the specificity of Smad3 antibody used for ChIP assay. **(C)** ChIP PCR shows the binding of Smad3 on the promoter of *E2F3* locus. The ChIP PCR product is analyzed by agarose electrophoresis. **(D)** RT-PCR is used to quantitate the ChIP efficiency of Smad3 antibody to precipitate *E2F3* promoter DNA compared with the non-specific IgG isotype, which is normalized to input.** (E)** The structure of recombined firefly luciferase expression vector driven by the *E2F3* promoter with normal or mutated Smad3 binding site. **(F)** Dual-luciferase reporter assay shows the transcriptional regulation of Smad3 on cloned *E2F3* promoter in HEK293T cells. Each dot represents one independent experiment and data are expressed as mean ± SD. *p < 0.05 and **p < 0.01 compared with control or as indicated.

To further confirm the predicted binding of Smad3 in the *E2F3* promoter, we performed chromatin immunoprecipitation (ChIP) in isolated mouse islets (Figure 10A). The specificity of the Smad3 antibody used for ChIP was initially validated by immunoprecipitation in mouse islets (Figure 10B). ChIP PCR confirmed the binding of Smad3 to the *E2F3* promoter as the anti-Smad3 antibody, but not non-specific IgG isotype successfully precipitated the chromatin fragment corresponding to the *E2F3* promoter (Figure 10C). Quantitative RT-PCR revealed a 6-fold enrichment of *E2F3* promoter sequence in the ChIP assay with the Smad3 antibody compared with the IgG isotype (Figure 10D), demonstrating that Smad3 can bind to the promoter region of *E2F3* genomic locus.

To validate the functional significance of Smad3 binding on the *E2F3* promoter, we constructed a luciferase reporter vector driven by mouse *E2F3* promoter harboring the Smad3 binding site or its mutant form (Figure 10E). Dual-luciferase assay in HEK293T cells revealed a robust transcription of luciferase driven by the cloned *E2F3* promoter, which was significantly inhibited by overexpressing Smad3. However, mutation of the Smad3 binding site abrogated the inhibitory effect by Smad3 (Figure 10F). These findings provided direct evidence for the suppressive role of Smad3 in *E2F3* transcription by binding to its promoter.

## Discussion

In this study, we demonstrated that transplantation of Smad3-deficient islets produced a better therapeutic effect on diabetes and diabetic kidney injury in STZ-induced diabetic mice and in db/db mice. In STZ-induced diabetes, transplantation of 100 Smad3KO but not 100 Smad3WT islets resulted in a 17-fold increase in β cell mass, which was associated with a significant increase in blood insulin levels and thus improved glucose intolerance and recovered euglycemia, resulting in protection against diabetic kidney injury.

Similar results were also found in T2DM in db/db mice in which transplantation with 250 Smad3KO islets but not the same dose of Smad3WT islets also increased a 5-fold β cell mass and produced a better glycemic control. These findings suggest that Smad3 deficiency is resistant to the diabetic microenvironments and largely promotes the expansion of grafted islet β cells and insulin production to exhibit better glycemic control. Interestingly, we also found that treatment with 100 Smad3KO islets could produce an equal glycemic control as the use of 200 Smad3WT or KO islets in STZ-induced diabetes. This observation suggests that Smad3 deficiency may be able to largely reduce (at least 50%) the number of islets required for the successful establishment of insulin independence in islet cell replacement therapy for T1DM. These findings keep in line with the previous report that pretreatment of the islets with SB-431542, an inhibitor of TGFBR1, promotes the proliferation and function of β cells after transplantation 15. However, as Smad2 also plays an essential role in the glucose-stimulated release of insulin in β cells 16, targeting Smad3 but not upstream TGFBR1 in islets (β cells) may be more favorable for cell replacement treatment of diabetes. Thus, Smad3-deficient islet transplantation may represent a novel therapy for both T1DM and T2DM clinically. In addition, treatment with Smad3KO islets could also protect against diabetic kidney injury at 16 weeks of T1DM, although this is not profound in T2DM due to the minimal diabetic kidney injury occurred in db/db mice at the age of week 12.

Mechanistically, we found that Smad3 functions by suppressing *E2F3* to inhibit β cell proliferation. This is supported by the evidence that the *E2F3* promoter contains a functional Smad3 binding site as confirmed by ChIP and luciferase reporter assay. Islet β cells lacking Smad3 showed enhanced proliferation in an E2F3-dependent mechanism as knockdown of E2F3 abolished this pro-proliferative activity. E2F3 belongs to the E2F family and is a critical component of the Rb-E2F machinery governing G1/S cell cycle entry 13. E2Fs are the transcriptional factors and final executors to regulate genes orchestrating cell cycle progress 14. Overexpression of E2F3 has been proved to promote β cell proliferation in islets from rodent and human 17. The findings that Smad3 transcriptionally targets *E2F3* to repress the cell cycle in β cells provide a novel regulatory mechanism for β cell proliferation. Thus, disruption of Smad3 in islet β cells promotes E2F3-dependent β cell proliferation as demonstrated by a marked increase in insulin-producing PCNA^+^ or BrdU^+^ β cells, suggesting that treatment with a subtherapeutic dose of Smad3KO islets is sufficient to expand β cell mass and inhibits both T1DM in STZ-induced mice and T2DM in db/db mice.

It is reported that TGF-β signaling plays a critical role in pancreatic islet β cell development and function, arrests the cell cycle, and causes β cell apoptosis by inducing CDKIs 7, 8, 18. To our surprise, both *in vivo* and *in vitro* experiments revealed that the transcription of CDKIs was not changed or even upregulated in Smad3KO islets (Figure S4). This discrepancy may be attributed to the different experimental conditions. The results in the current study were derived from Smad3KO mice (islets). However, the previous studies are based on the pharmacological inhibition of TGFBR1, which shall influence the entire TGF-β signaling 7, 8. Furthermore, we used islet samples from young adult mice (age of 8-10 weeks), while islets from relatively old animals or cadaveric donors of aged people were used in previous studies 7, 8. As islet β cells show age-associated accumulation of CDKIs 19, CDKIs in aged β cells may be more sensitive to TGF-β signaling inhibition.

There were several limitations in the present study. First, the efficacy of islet transplantation is investigated in the STZ-induced diabetic mouse model without autoimmune reaction. Therefore, the advantage of Smad3KO over Smad3WT islet therapy for diabetes should also be verified in an authentic T1DM model in the future. Second, the findings from this study are based on the whole-islet but not β cell-specific Smad3KO cell therapy. It's unclear whether cell types other than β cells in the islet would execute a Smad3-dependent role to modulate β cell proliferation by intercellular crosstalk pathways. Thus, further studies with β cell specific Smad3KO are warranted. Furthermore, findings from this study need to be verified in human islet samples to prove their clinical significance.

## Conclusions

Smad3 deficiency largely improves islet replacement therapy for both T1DM and T2DM and protects against diabetic kidney injury. Enhanced E2F3-dependent β cell proliferation may be a key mechanism of Smad3KO islet therapy in both T1DM and T2DM. Thus, Smad3KO β cell replacement therapy may be a better therapeutic strategy for diabetes clinically.

## Methods

### Animals

The Smad3-null (Smad3KO) mice are a kind gift from Prof. Chuxia Deng and maintained in heterozygotes (Smad3^+/-^) in C57BL/6 background 20. Smad3KO mice were generated by intercrossing of Smad3^+/-^ mice. The db/db mice with mutation of *Lepr* (Leptin receptor) in C57BLKS background were purchased from the Animal Experimental Center of the Chinese University of Hong Kong (CUHK). All animals were maintained in a specific pathogen-free (SPF) facility with free access to food and water at a day/night cycle of 12/12 h. All animal manipulations in this study were performed following the regulations of and approved by the Ethics Committee of Animal Experiment of CUHK with the Ref. No. 18-177-MIS.

### Streptozocin (STZ)-induced diabetic mouse model

Male C57BL/6 mice at age of 8-10 weeks were intraperitoneally injected with 50 mg/kg/day STZ daily for 5 continuous days. 5 days post the final STZ injection, mice with a stable random blood glucose of more than 16 mM for 2 consecutive days were regarded as the successful establishment of diabetes and used as the recipient mice for islet transplantation.

### Islet isolation, transplantation, and assessment of diabetic phenotype

Islets were isolated from male or female Smad3 knockout (KO) or wild-type (WT) mice at the age of 8-10 weeks as previously described 21. To perform the islet transplantation, indicated number of islets were transplanted under the renal capsule of male db/db mice at the age of week 4 or STZ-induced diabetic mice on day 7 post the final STZ injection following the protocol described by Szot et al 22. Briefly, the recipient mouse was anesthetized by the anesthesia Combo (1.5% ketamine and 0.15% xylazine, 6 μL/g bodyweight). After the loss of consciousness, an incision of 1 cm was made on the skin covering the left kidney. Pull out the kidney gently and keep it moist with sterile saline during the surgery. With a 25G needle, make a small scratch on the capsule to create a nick. Indicated numbers of islets were delivered into the renal capsule by a PE50 tubing. After all islets were transplanted, seal the nick by cauterization with low heat. Replace the kidney and close the peritoneum with sutures. Seal the skin with staples. 100 μL Penicillin-streptomycin solution (Gibco, cat# 15140122, USA) was injected intraperitoneally to prevent infection. 100 μL Tegmic was injected intramuscularly to relieve the pain. The animal was placed in a warm environment until full recovery. The same operation procedure was also carried in sham control mice but without islet infusion. Both random and 6 h-fasting blood glucose (RBG and FBG) levels of the tail tip blood were checked weekly with a portable glucose meter (Roche, ACCU-CHEK® Performa). The insulin resistance test (IPITT), glucose tolerance test (IPGTT), levels of HbA1c, and serum insulin were measured as previously described 9.

### Total urinary protein and serum creatinine

24 h urine was collected in metabolic cage. 24 h total urinary protein was calculated by multiplying the urine volume and urinary protein concentration which was quantitated by the Quick Start Bradford Protein Assay Kit (Bio-Rad, cat# 5000201, USA). Serum creatinine was determined with the creatinine quantification kit (Stanbio Laboratory, cat# 0430-120, USA).

### Histopathology

Renal histopathological changes were examined in paraformaldehyde-fixed, paraffin-embedded sections (4 µm) with Periodic Acid-Schiff's (PAS) reagent as previously described 23. Mesangial matrix expansion was scored using the quantitative image program (Image-Pro Plus 7.0, Media Cybernetics) as previously described 23.

### Immunohistochemistry

Islets-bearing kidney or pancreas was fixed in 4% paraformaldehyde, embedded in paraffin, and sectioned at 4 μm. After rehydration, the sections were treated with microwave-mediated antigen retrieval in 0.01 M citrate (pH 6.0) for 10 min and blocking in 5% BSA for 1 h. Primary antibody incubation was performed overnight at 4 °C. For fluorescent staining, a fluorescent secondary antibody was applied at room temperature (RT) for 1 h. For DAB-mediated chromogenesis, an additional step of 3% H_2_O_2_ blocking for 15 min was included before the antigen retrieval. After primary antibody incubation, HRP-conjugated polymer (Envision+ System, Dako, USA) was added. For staining of E2F3, antigen retrieval was performed in EDTA-Tris solution (pH 9.0). Antibodies used in immunohistochemistry were detailed in Table S2. The staining-positive area was quantitated with Image J software (v1.47, NIH, USA).

### Measurement of grafted β cell mass in the islets-bearing kidney

An index of average β cell area per section was used to semi-quantitatively quantify the β cell mass in islet graft. Briefly, the islets-bearing kidney (paraffin-embedded) was sectioned successively at 4 μm along the sagittal axis. Continuous sections at 160 μm intervals were fluorescently immunostained for insulin. At least 6 sections with insulin-positive staining were used for the calculation of β cell area per section in each mouse. The insulin-positive β cell area in each section was quantified with Image J software (v1.47, NIH, USA). The index of average β cell area per section in each mouse was calculated by dividing the total insulin-positive area in all sections by the number of insulin-positive sections.

### Primary culture of islet cells

The isolated islets were dissociated into single cells and seeded into the 96-well plate in 200 μL of RPMI 1640 (Gibco, USA) + 10% FBS (Gibco, USA) + 1% Penicillin-streptomycin (Gibco, USA) supplemented with 20 mM glucose. Islet cells were cultured for indicated time in an incubator set at 37 °C, 5% CO_2_, and 100% humidity. For adenovirus-mediated knockdown of *E2F3*, dissociated islet cells were cultured in medium containing adenovirus overexpressing short hairpin RNA (shRNA) targeting mouse *E2F3* (shE2F3) or control non-specific shRNA (shCon) at a titer of 10 pfu per cell for 2 days followed by medium replacement. The stem sequence of the shE2F3 duplex was 5'-CATCCATGCTCTATTCTGT-3'. Dispersed cells from 50 islets were seeded per well for immunostaining and RT-PCR, and 100 islets per well for Western blot, which were conducted as previously described 9.

### *In vivo* and *in vitro* BrdU labeling

To perform *in vivo* BrdU labeling, the mice were intraperitoneally injected with 50 mg/kg/day BrdU (in PBS) for 7 days from the 2nd day after islet transplantation and sacrificed 2 h after the last injection. For *in vitro* BrdU labeling, the dispersed islet cells were cultured for 48 h followed by replacement with fresh medium containing 10 μM BrdU and cultured for additional 24 h. Fluorescent immunostaining against BrdU in the tissue and cultured cells was performed as previously described 9.

### RNA-Seq

Islets were isolated from Smad3WT-db/db, Smad3KO-db/db, Smad3WT-db/m, and Smad3KO-db/m mice. For each genotype, islets from more than 5 mice were pooled for RNA isolation. RNA was prepared with the miRNeasy Mini Kit (Qiagen, Germany). RNA-seq and the downstream data analysis were described previously 9. The expression level of a given gene was presented as the fragments per kilobase of transcript per million mapped reads (FPKM). Differentially expressed gene (DEG) was defined as ≥ 2 folds variance of FPKM between two groups. Upregulated and downregulated DEGs were combined and used for Gene Ontology (GO) and Kyoto Encyclopedia of Genes and Genomes (KEGG) pathway enrichment analysis with the online DAVID bioinformatics resources (v6.8) with default settings. The GOTERM_BP_DIRECT sub-category was employed for GO analysis.

### Immunoprecipitation

To perform immunoprecipitation in mouse islets, about 500 freshly-isolated islets were lysed in 500 mL of RIPA buffer. A rabbit anti-Smad3 antibody (2 μg) or rabbit IgG isotype was added and incubated at 4 °C for overnight with gentle rotation. Then, 20 μL of protein A agarose beads (Beyotime, cat# P2051, China) was added and incubated for 2 h at 4 °C. After washing robustly with fresh RIPA buffer, the bounded protein was released by denaturing in 1xSDS loading buffer and subjected to Western blot analysis. To avoid the influence of IgG heavy chain, a light chain-specific mouse anti-rabbit IgG (HRP conjugated, Abmart, cat# M21006, China) was used to recognize the precipitated Smad3 protein.

### Chromatin immunoprecipitation-ChIP

To perform ChIP in mouse islets, about 2000 islets were collected from 8-10 weeks old C57BL/6 mice and used as one ChIP preparation. The freshly isolated islets were stimulated with 10 ng/mL TGF-β1 (Gibco, USA) for 15 min in RPMI 1640 + 1% FBS +1% Penicillin-streptomycin. ChIP was performed with the SimpleChIP® Plus Enzymatic Chromatin IP Kit (CST, USA) following the recommended instruction. Antibody and primers used in the ChIP assay are detailed in Table S1 and S2.

### Dual-luciferase reporter assay

Mouse *E2F3* promoter (-1000 to +451 bp) was cloned into the pGL3-basic vector (pGL3-E2F3.Pro). Concurrently, point mutation of the Smad3 binding site was conducted to generate the pGL3-E2F3.Pro.mut. To perform the dual-luciferase reporter assay, HEK293T cells were seeded into the 24-well plate at a density of 5 x 10^4^ cells/well. The next day, cells were transfected with 500 ng pGL3-Basic or pGL3-E2F3.pro or pGL3-E2F3.pro.mut combined with pcDNA3.1-Smad3, which overexpresses mouse Smad3, or not (as indicated in Figure 10F) by phosphate calcium method. The pEGFP-C1 vector was supplemented to balance the transfection dose in each treatment. 10 ng pGL4.73.hRluc/SV40 vector expressing renilla luciferase was included in each treatment to normalize the transfection efficiency. 6 h post-transfection, the medium was changed followed by incubation for additional 48 h. The cells were lysed, and luciferase activity was determined with the Dual-Luciferase ® Reporter Assay System (Promega, USA) and quantified in a luminometer (VICTOR^TM^ X4 2030 Multilable Reader, PerkinElmer, USA). The transcriptional activity of the cloned E2F3 promoter was presented as the value ratio of firefly luciferase to renilla luciferase. Each treatment was performed in triplicates.

### Statistics

Data were presented as mean ± SD and analyzed with SPSS software (16.0). The normality of the data was checked by One-Sample Kolmogorov-Smirnov test. The homogeneity of variances among groups was tested by the Levene statistic. For comparison between two groups, Student's t test was used. For comparison among multiple groups, one-way ANOVA with LSD test was used if the equal variance was assumed. For the equal variance wasn't assumed, an alternative Welch test statistic was used followed by multiple comparisons with Tamhane's T2 test. *P* < 0.05 was considered statistically significant.

## Supplementary Material

Supplementary figures and tables.

## Abbreviations

ANOVA: analysis of variance
BrdU: 5-bromo-2'-deoxyuridine
CDKs: cyclin-dependent kinases
CDKIs: cyclin-dependent kinase inhibitors
ChIP: chromatin immunoprecipitation
DAB: 3,3'-diaminobenzidine
DEG: differentially expressed gene
E2F3: E2F transcription factor 3
FBG: fasting blood glucose
HA1c: hemoglobin A1c
IPGTT: intraperitoneal glucose tolerance test
IPITT: intraperitoneal insulin tolerance test
iPSC: induced pluripotent stem cells
KO: knock-out
PAS: periodic acid Schiff
RBG: random blood glucose
RT: room temperature
STZ: streptozocin
T1DM: type-1 diabetes mellitus
T2DM: type-2 diabetes mellitus
TGFBR: TGF-β receptor
TGF-β: transforming growth factor-β
WT: wild-type

## References

[1] Shapiro AM, Pokrywczynska M, Ricordi C (2017). Clinical pancreatic islet transplantation. Nat Rev Endocrinol.

[2] Jacobson EF, Tzanakakis ES (2017). Human pluripotent stem cell differentiation to functional pancreatic cells for diabetes therapies: Innovations, challenges and future directions. J Biol Eng.

[3] Toso C, Isse K, Demetris AJ, Dinyari P, Koh A, Imes S (2009). Histologic graft assessment after clinical islet transplantation. Transplantation.

[4] Lan HY (2011). Diverse roles of TGF-beta/Smads in renal fibrosis and inflammation. Int J Biol Sci.

[5] Meng XM, Tang PM, Li J, Lan HY (2015). TGF-beta/Smad signaling in renal fibrosis. Front Physiol.

[6] El-Gohary Y, Tulachan S, Wiersch J, Guo P, Welsh C, Prasadan K (2014). A smad signaling network regulates islet cell proliferation. Diabetes.

[7] Dhawan S, Dirice E, Kulkarni RN, Bhushan A (2016). Inhibition of TGF-beta Signaling Promotes Human Pancreatic beta-Cell Replication. Diabetes.

[8] Wang P, Karakose E, Liu H, Swartz E, Ackeifi C, Zlatanic V (2019). Combined Inhibition of DYRK1A, SMAD, and Trithorax Pathways Synergizes to Induce Robust Replication in Adult Human Beta Cells. Cell Metab.

[9] Sheng J-Y, Wang L, Tang PM-K, Wang H-L, Li J-C, Xu B-H (2021). Smad3 deficiency promotes beta cell proliferation and function in db/db mice via restoring Pax6 expression. Theranostics.

[10] Zmuda EJ, Powell CA, Hai T (2011). A method for murine islet isolation and subcapsular kidney transplantation. J Vis Exp.

[11] Choi MY, Lim SJ, Kim MJ, Wee YM, Kwon H, Jung CH (2021). Islet isograft transplantation improves insulin sensitivity in a murine model of type 2 diabetes. Endocrine.

[12] Vijayachandra K, Higgins W, Lee J, Glick A (2009). Induction of p16ink4a and p19ARF by TGFbeta1 contributes to growth arrest and senescence response in mouse keratinocytes. Mol Carcinog.

[13] Dyer MA, Cepko CL (2001). Regulating proliferation during retinal development. Nat Rev Neurosci.

[14] Dyson N (1998). The regulation of E2F by pRB-family proteins. Genes Dev.

[15] Xiao X, Fischbach S, Song Z, Gaffar I, Zimmerman R, Wiersch J (2016). Transient Suppression of TGFbeta Receptor Signaling Facilitates Human Islet Transplantation. Endocrinology.

[16] Nomura M, Zhu HL, Wang L, Morinaga H, Takayanagi R, Teramoto N (2014). SMAD2 disruption in mouse pancreatic beta cells leads to islet hyperplasia and impaired insulin secretion due to the attenuation of ATP-sensitive K+ channel activity. Diabetologia.

[17] Rady B, Chen Y, Vaca P, Wang Q, Wang Y, Salmon P (2013). Overexpression of E2F3 promotes proliferation of functional human beta cells without induction of apoptosis. Cell Cycle.

[18] Zhang Y, Alexander PB, Wang XF (2017). TGF-beta Family Signaling in the Control of Cell Proliferation and Survival. Cold Spring Harb Perspect Biol.

[19] Wang P, Fiaschi-Taesch NM, Vasavada RC, Scott DK, Garcia-Ocana A, Stewart AF (2015). Diabetes mellitus-advances and challenges in human beta-cell proliferation. Nat Rev Endocrinol.

[20] Yang X, Letterio JJ, Lechleider RJ, Chen L, Hayman R, Gu H (1999). Targeted disruption of SMAD3 results in impaired mucosal immunity and diminished T cell responsiveness to TGF-beta. EMBO J.

[21] Li DS, Yuan YH, Tu HJ, Liang QL, Dai LJ (2009). A protocol for islet isolation from mouse pancreas. Nat Protoc.

[22] Szot GL, Koudria P, Bluestone JA Transplantation of pancreatic islets into the kidney capsule of diabetic mice. J Vis Exp. 2007: 404.

[23] Xu BH, Sheng J, You YK, Huang XR, Ma RCW, Wang Q (2020). Deletion of Smad3 prevents renal fibrosis and inflammation in type 2 diabetic nephropathy. Metabolism.

