# Supplementary material for: Smad3 deficiency improves islet-based therapy for diabetes and diabetic kidney injury by promoting β cell proliferation via the E2F3-dependent mechanism
Source: Theranostics. 2022 Jan 1;12(1):379–95. doi: 10.7150/thno.67034 (PMC8690916; doi:10.7150/thno.67034)
Supplement: Supplementary file 1 — Supplementary figures and tables. [file thnov12p0379s1.pdf]

Smad3 deficiency improves islet-based therapy for diabetes and diabetic kidney injury by promoting β cell proliferation *via* the E2F3-dependent mechanism

Hong-Lian Wang[1,2], Biao Wei[2] Hui-Jun He[2], Xiao-Ru Huang[2,3], Jing-Yi Sheng[2,4], Xiao-Cui Chen[2,5], Li Wang[1], Rui-Zhi Tan[1], Jian-Chun Li[1], Jian Liu[1], Si-Jin Yang[6], Ronald CW Ma[2], and Hui-Yao Lan[2,7]*

[1]Research Center for Integrated Medicine and Department of Nephrology, Affiliated Traditional Chinese Medicine Hospital of Southwest Medical University, Luzhou, Sichuan, 646000, China.
[2]Department of Medicine and Therapeutics, and Li Ka Shing Institute of Health Sciences, the Chinese University of Hong Kong, Hong Kong, 999077, China.
[3]Guangdong-Hong Kong Joint Laboratory on Immunological and Genetic Kidney Diseases, Guangdong Provincial People's Hospital, Guangdong Academy of Medical Sciences, Guangzhou, Guangdong, 510080, China.
[4]State Key Laboratory of Bioelectronics, Jiangsu Key Laboratory for Biomaterials and Devices, School of Biological Sciences & Medical Engineering, Southeast University, Nanjing, China
[5]Key Laboratory of Prevention and Management of Chronic Kidney Disease of Zhanjiang City, Institute of Nephrology, Affiliated Hospital of Guangdong Medical University, Zhanjiang, Guangdong, 524001, China
[6]National Traditional Chinese Medicine Clinical Research Base, Affiliated Traditional Chinese Medicine Hospital of Southwest Medical University, Luzhou, Sichuan, 646000, China
[7]CUHK-Guangdong Provincial People's Hospital Joint Research Laboratory on Immunological and Genetic Kidney Diseases, the Chinese University of Hong Kong, Hong Kong, 999077, China

***Correspondence** to Prof. Hui-Yao Lan, Li Ka Shing Institute of Health Sciences, the Prince of Wales Hospital, Shatin, Hong Kong SAR, China. Address: Prince of Wales Hospital, Shatin, N.T. Hong Kong SAR, China. Tel: +852-37636061, E-mail: hylan@cuhk.edu.hk.

Supplementary Figures and Tables

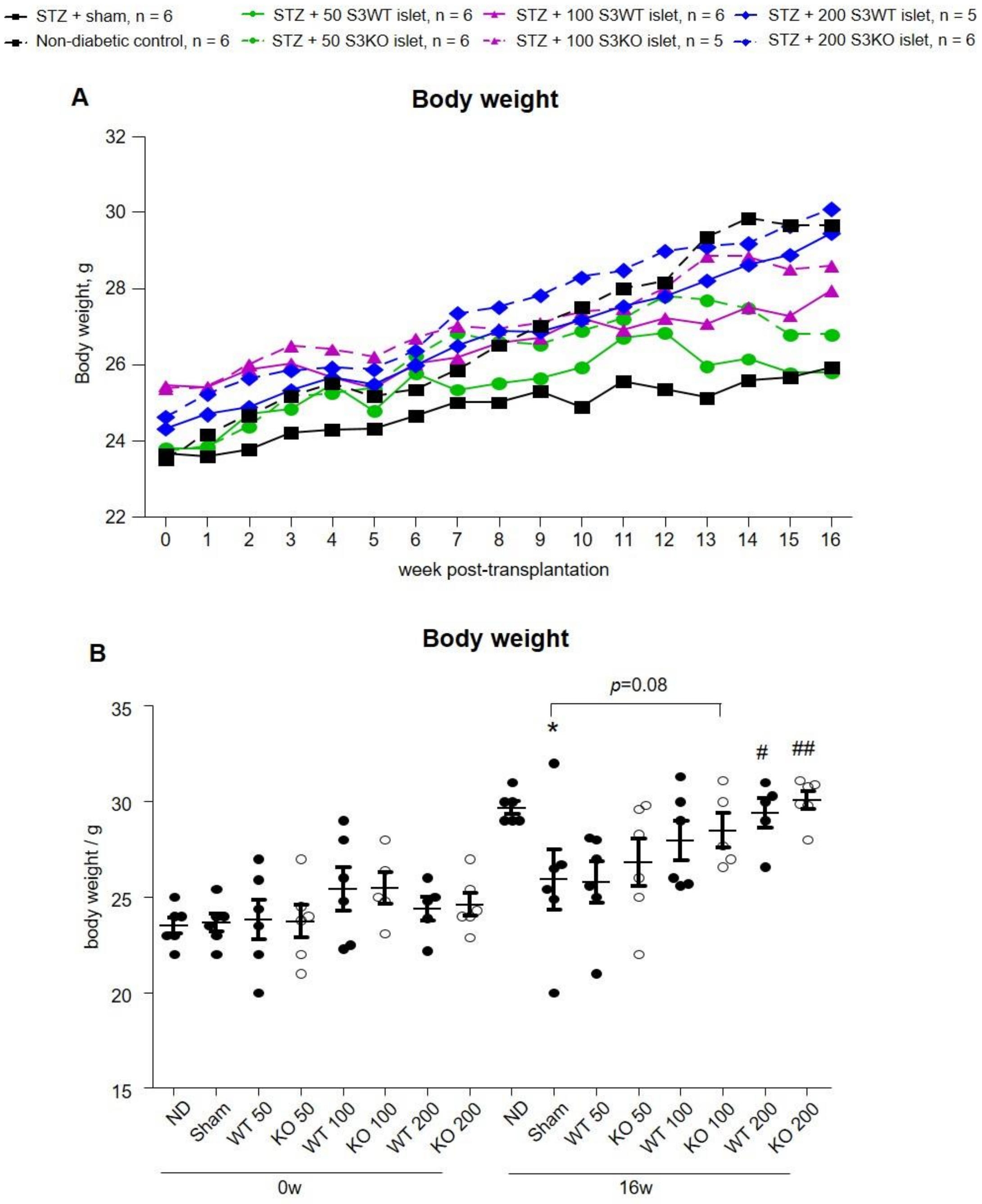

Figure S1. Effect of Smad3KO or WT islet transplantation on body weight in STZ-induced diabetic mice. (**A**) The mean value of weekly body weight over 16 weeks. **(B)** Body weight of mice before (0 week) and after islet therapy at week 16. Each dot represents one animal. ND, non-diabetic normal control. *$p < 0.05$ versus the age-matched normal control (ND). $^{\#}p < 0.05$ and $^{\#\#}$ $p < 0.01$ versus the age-matched Sham group.

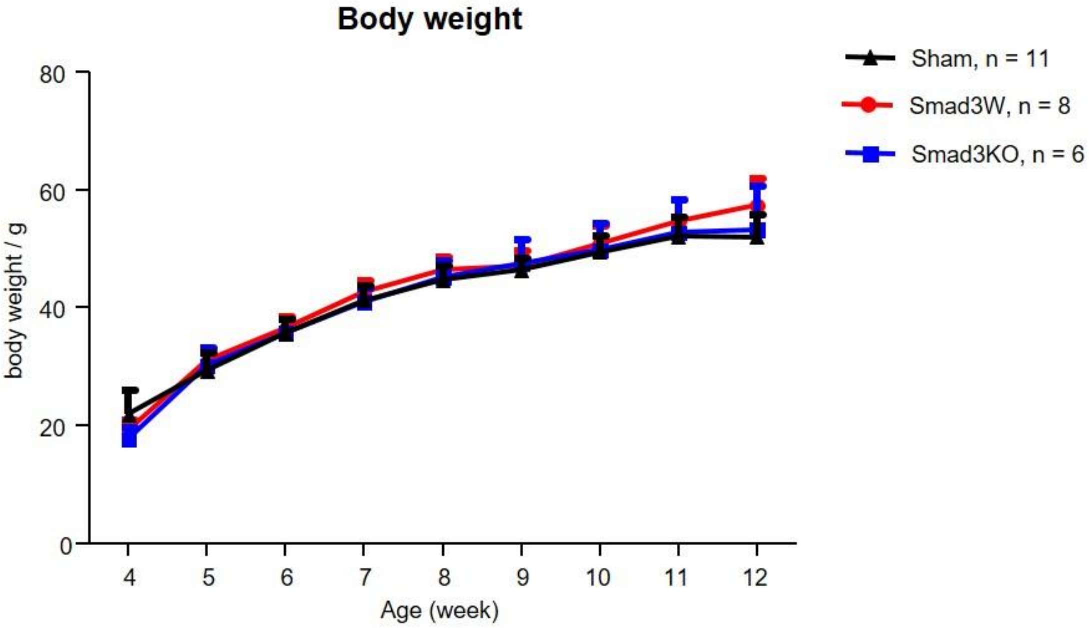

Figure S2. Transplantation of Smad3KO or Smad3WT islets doesn't influence the gain of body weight of recipient db/db mice.

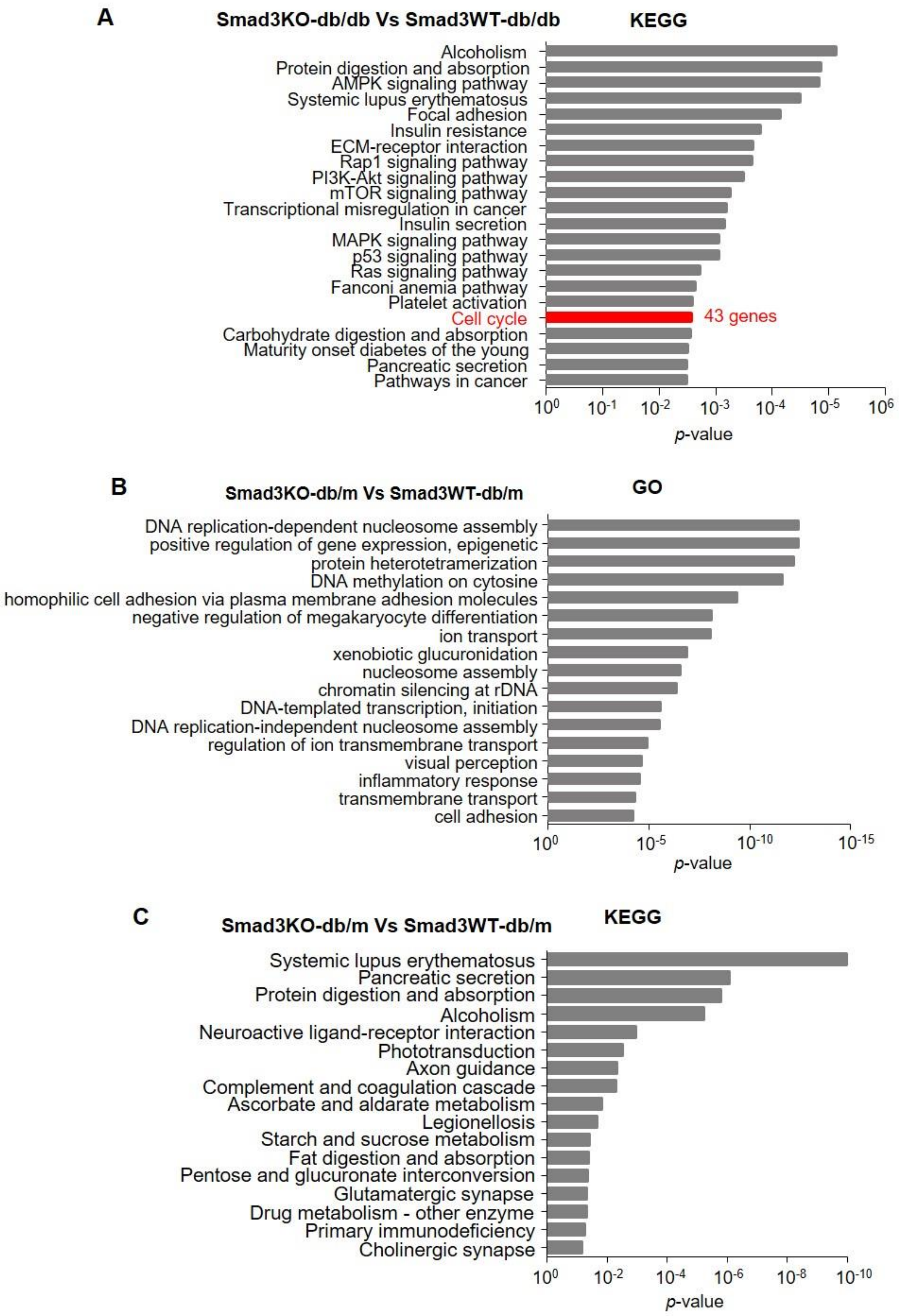

Figure S3. Bioinformatic analysis of DEGs in islets by RNA-seq. (A) KEGG pathway analysis of islet DEGs between Smad3WT-db/db and Smad3KO-db/db. (**B** and **C)** GO (GOTERM_BP_DIRECT subcategory) and KEGG analysis of DEGs in islets between Smad3WT-db/m and Smad3KO-db/m.

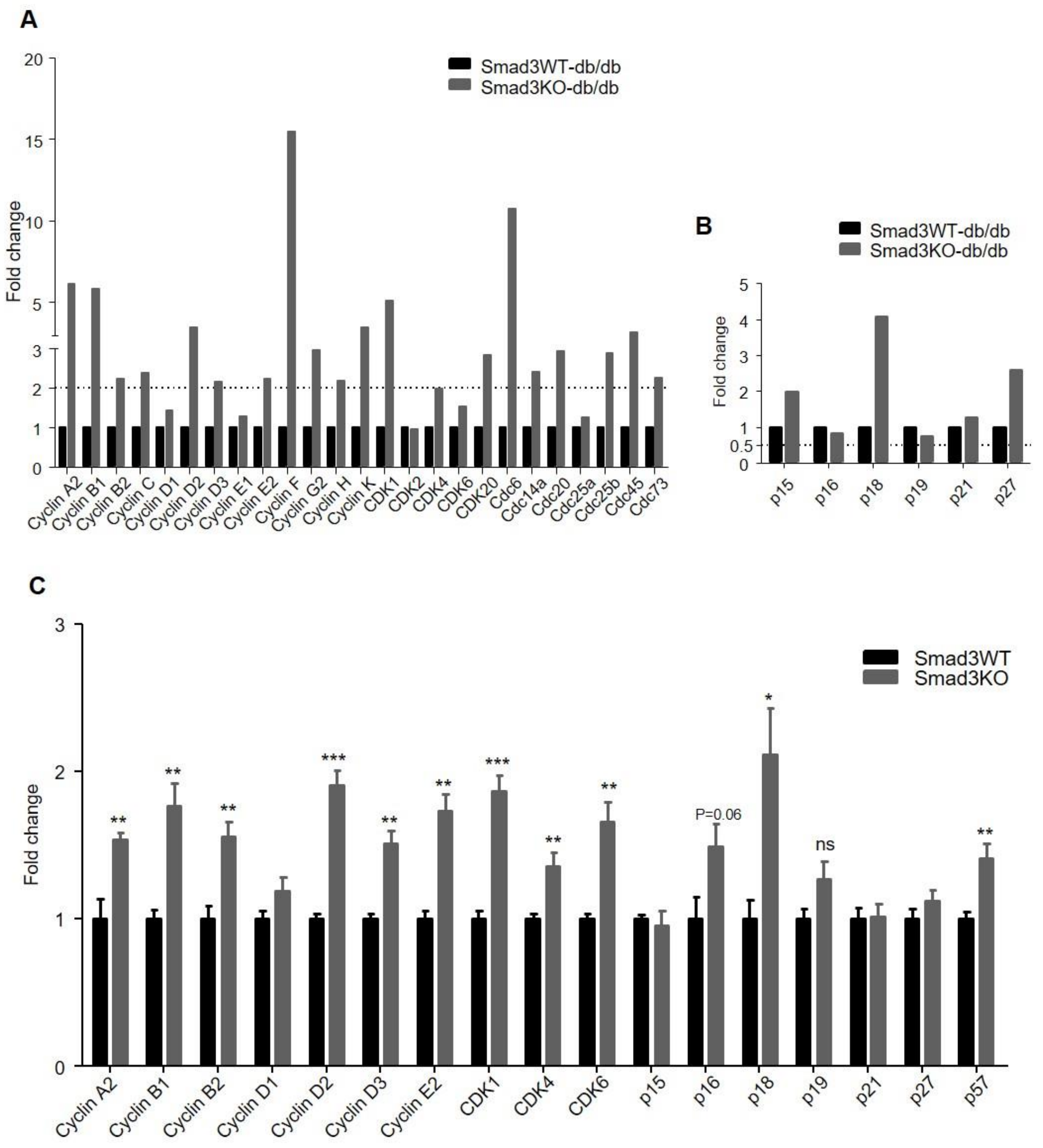

Figure S4. Expression of cell cycle-related genes in the islets isolated from Smad3WT-db/db and Smad3KO-db/db mice, and in cultured Smad3WT and Smad3KO islet cells. (A) Relative expression of genes associated with cyclin, CDK and CDC family revealed by RNA-seq. (**B)** Relative expression of genes from CDKI family revealed by RNA-seq. **(C)** RT-PCR detects the expression of selected cell cycle-related genes in 48 h-cultured islet cells. n = 4 culture replicates for each group. *$p < 0.05$, **$p < 0.01$ and ***$p < 0.001$ versus Smad3WT. ns, no significance.

Table S1. List of primers used in this study.

Primer name	Sequence (forward/reverse)	Application
E2f3.ChIP RT-PCR	CATGAAGCTGCCAGGAATGA	ChIP RT-PCR
	TCCAACCAATGACGTGAGAG	
E2f3.ChIP PCR	CAGAGAGGGTGCTTGTTAGA	ChIP PCR
	GTGTCAAAGGCAAGTTGGAC	
RT.β-actin	AGAGGGAAATCGTGCGTGAC	RT-PCR
	CAATAGTGATGACC TGGCCGT	
RT.E2F3	AAGTCCTGAGATGCAGGTTC	RT-PCR
	TGAGCGGACTGTATCGACTT	
RT.Cyclin A2	CTTACCCAGTACTTCCTGCA	RT-PCR
	CTCTGTCCTGTGACTGTGTA	
RT.Cyclin B1	AGGCCGTGACAAAGGCATAA	RT-PCR
	GCCTAAACTCAGAAGCAACAACA	
RT.Cyclin B2	TTCTCTGATGCTCTGCTCTG	RT-PCR
	ACAGAGTTTCCTGCAGAAGC	
RT.Cyclin D1	ATCTGCATCACCCTGAGAGT	RT-PCR
	GCTGTGGCTTTTCAGCAAAG	
RT.Cyclin D2	GTTCCTAAGAACCAGGCAGC	RT-PCR
	GGTCACTTACCCTGCTAGCA	
RT.Cyclin D3	CTGCTCTTAGAGGGAACAGCC	RT-PCR
	GCACCCTTAAGACCCCACAA	
RT.Cyclin E2	GCTTTGGCTTCTTCTACTCG	RT-PCR
	GTTGACACAGTCCTCATCTC	
RT.CDK1	CAACAGAACCATCGCACTGA	RT-PCR
	AGCAGGCTGTTTAGAGGCTA	
RT.CDK4	CTTTGCAGAGATGTTCCGTC	RT-PCR
	GGTCAGCATTTCCAGTAGCA	
RT.CDK6	AGCTTCTAGAGGCTTCAGCA	RT-PCR
	GGTAAGATGCAGGAAGATGC	
RT.p15	GAAGACTGCAAGCACGAAGA	RT-PCR
	AGCTGCAGAAAATGCGTAGG	
RT.p16	GACATCAAGACATCGTGCGA	RT-PCR
	GGATTTAGCTCTGCTCTTGG	
RT.p18	CCTTATGAAGCACACAGCCT	RT-PCR
	CACATTCACTGCAGGCTTGT	
RT.p19	CTAGAGTTCTGATCCAGCTC	RT-PCR
	CCAGAAGCATAGTGGATACC	
RT.p21	AGGCATATCTAGGCACTTGC	RT-PCR
	CCACACACCATAGAATGCTC	
RT.p27	TCTCAGGCAAACTCTGAGGA	RT-PCR
	AGAATCTTCTGCAGCAGGTC	
RT.p57	GCAAACGTCTGAGATGAGTT	RT-PCR
	TCCTTGCACATGGTACAGAG	

Table S2. List of antibodies used in this study.

Antibody	Company	Catalog No.	Application
	**Primary antibody**		
Rabbit anti-Smad3 ChIP grade	Abcam, USA	ab28379	ChIP, IP
Rabbit anti-PCNA	Santa Cruz	Sc-7907	WB, F-IHC
Rabbit anti-E2F3	Abcam	ab50917	WB, IHC
Mouse anti-BrdU	Dako	M0744	F-IHC
Alexa Fluor 488-conjugated insulin monoclonal antibody	eBioscience	53-9769-82	F-IHC, F-ICC
Rabbit anti-insulin	Abcam	ab181547	F-IHC, F-ICC
Rabbit anti-Col1a1	Cell Signaling,	72026	IHC
	**Secondary antibody**		
DyLight^{TM} 800 conjugated goat anti-rabbit IgG (H&L)	RockLand, USA	611-145-002	WB
DyLight^{TM} 680 conjugated sheep anti-mouse IgG (H&L)	RockLand, USA	610-644-002	WB
DyLight^{TM} 800 conjugated rabbit anti-mouse IgG (H&L)	RockLand, USA	610-445-002	WB
Rhodamine-conjugated Goat Anti-Mouse IgG Antibody	Merck Minipore	AP124R	F-IHC, F-ICC
Donkey anti-Rabbit IgG Secondary Antibody, Alexa Fluor 488	Thermo Scientific	A21206	F-IHC, F-ICC
Mouse anti-Rabbit IgG HRP (Light-chain specific)	Abmart	M21006	WB (after IP)

Note: ChIP, chromatin immunoprecipitation. WB, Western blot. IHC, immunohistochemistry. F-IHC, fluorescent immunohistochemistry. F-ICC, fluorescent immunocytochemistry. IP, immunoprecipitation.
